# Supplementary material for: Novel UHRF1-MYC Axis in Acute Lymphoblastic Leukemia
Source: Cancers (Basel). 2022 Aug 31;14(17):4262. doi: 10.3390/cancers14174262 (PMC9455066; doi:10.3390/cancers14174262)

Uncut WB for Supplemental Figure S1.

Figure S1.

C

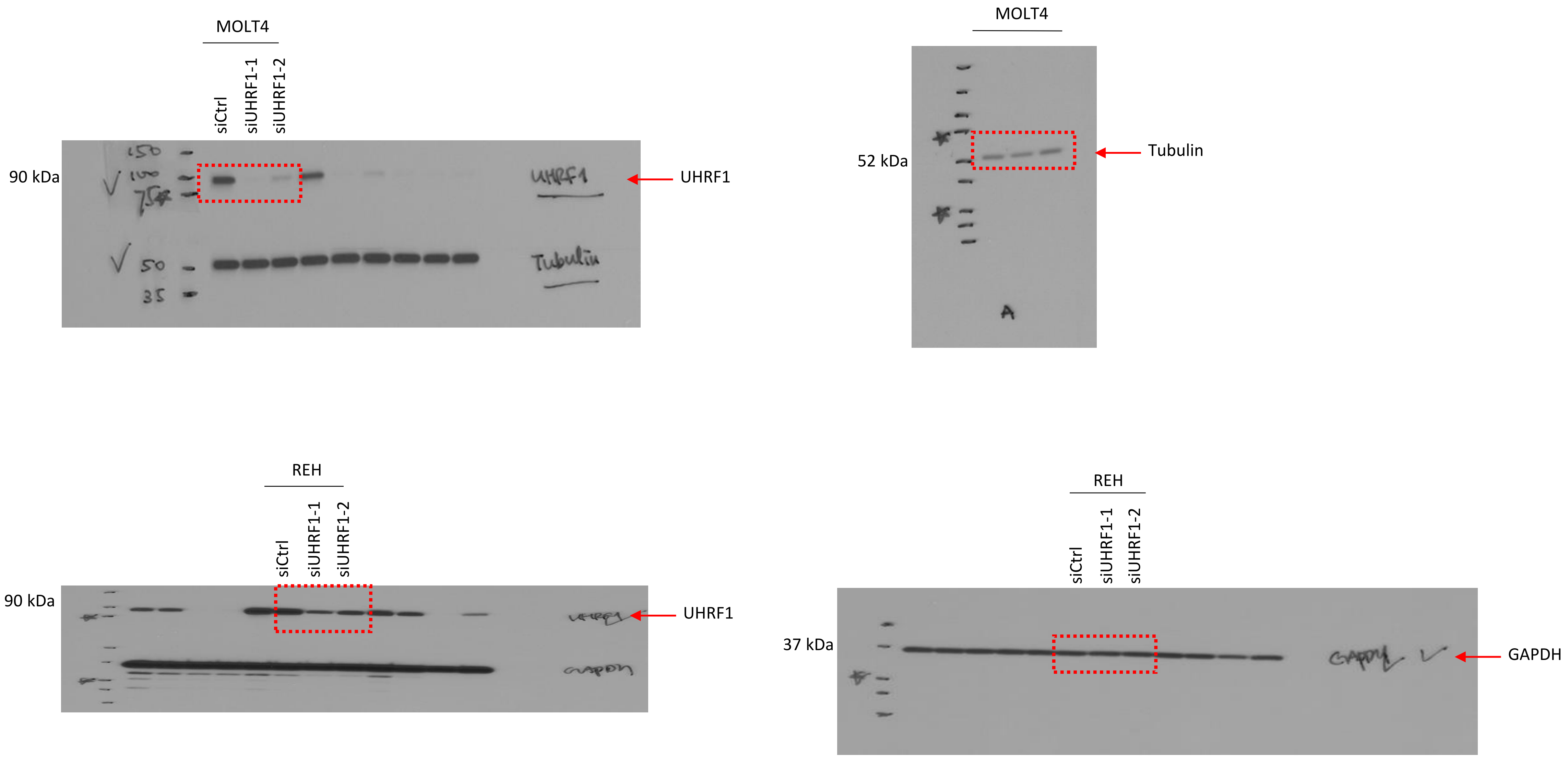

Figure 3 A and B.

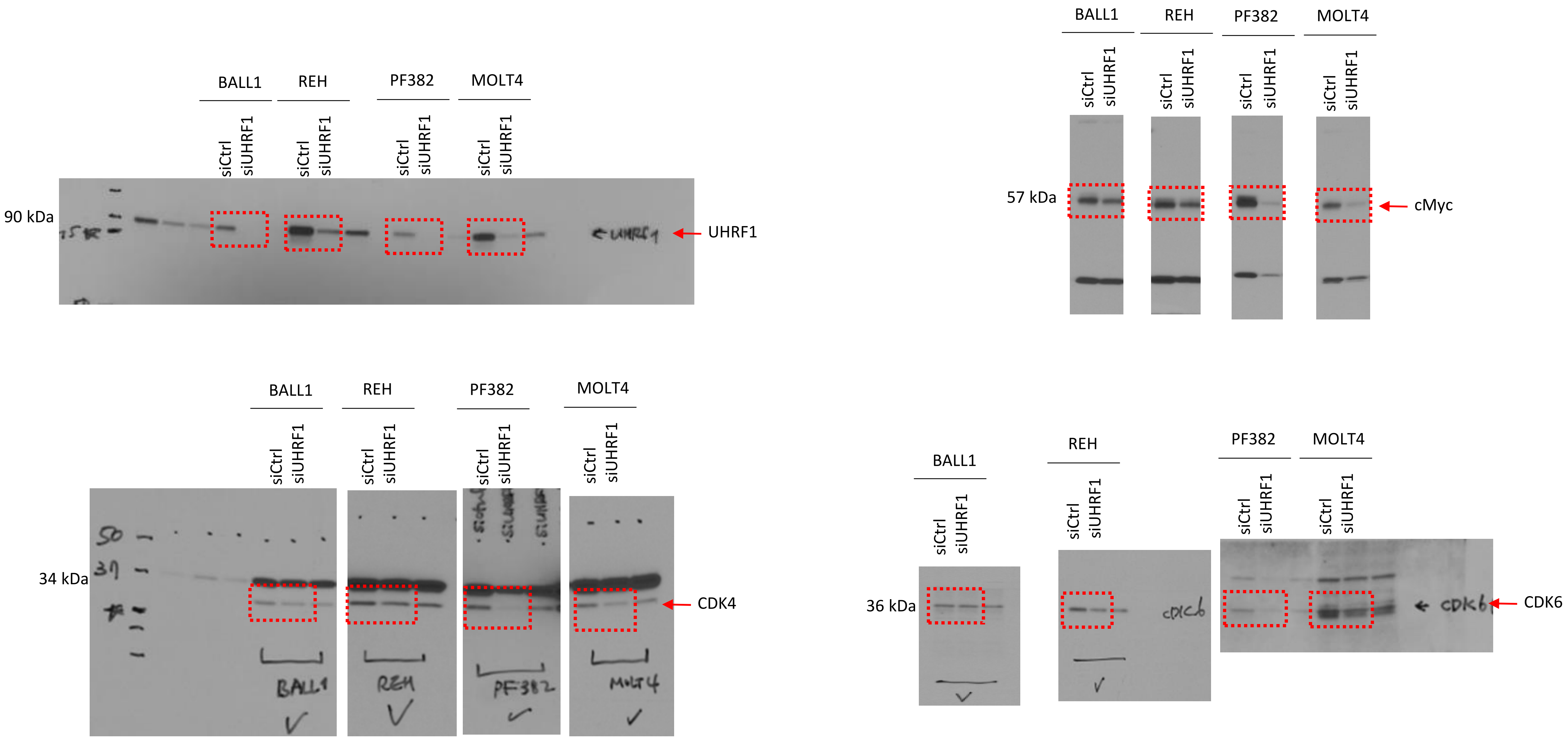

Figure 3 A and B.

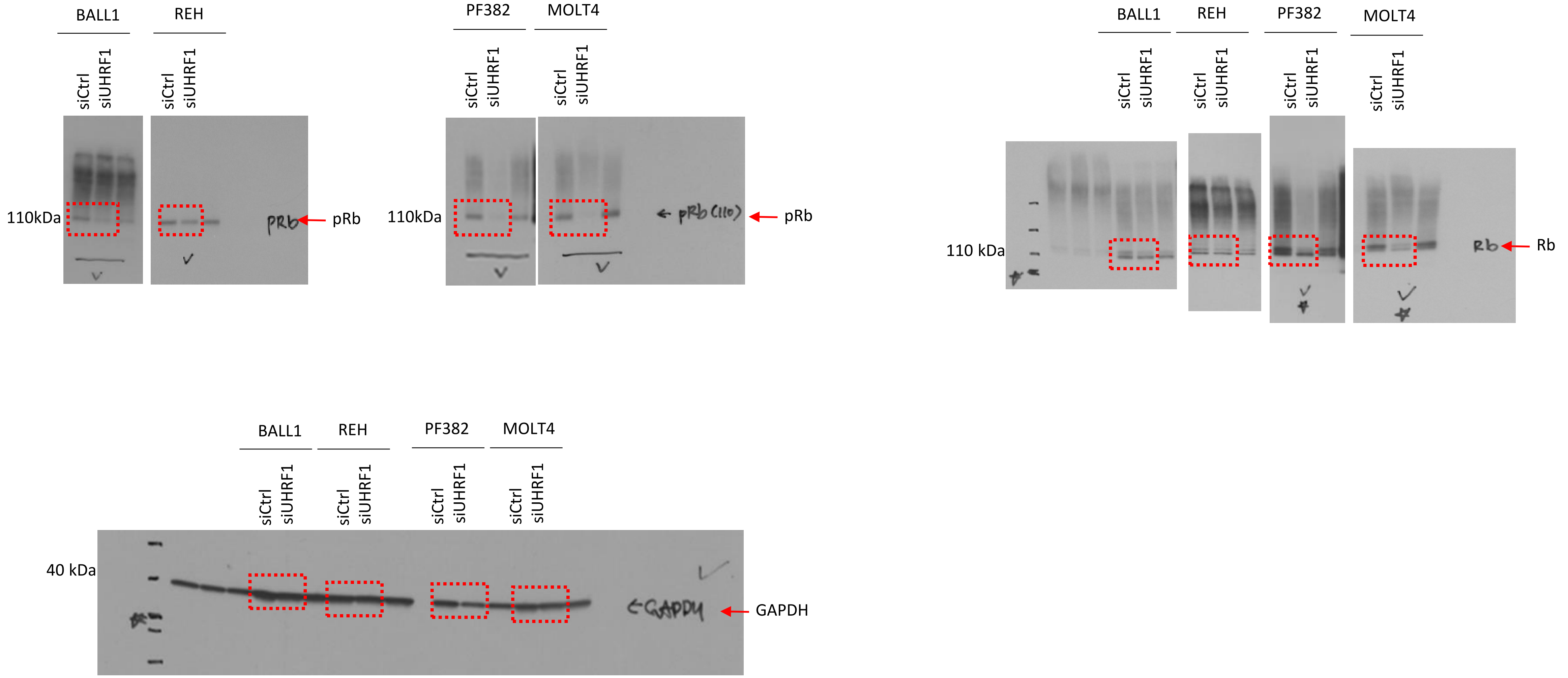

Figure 4.

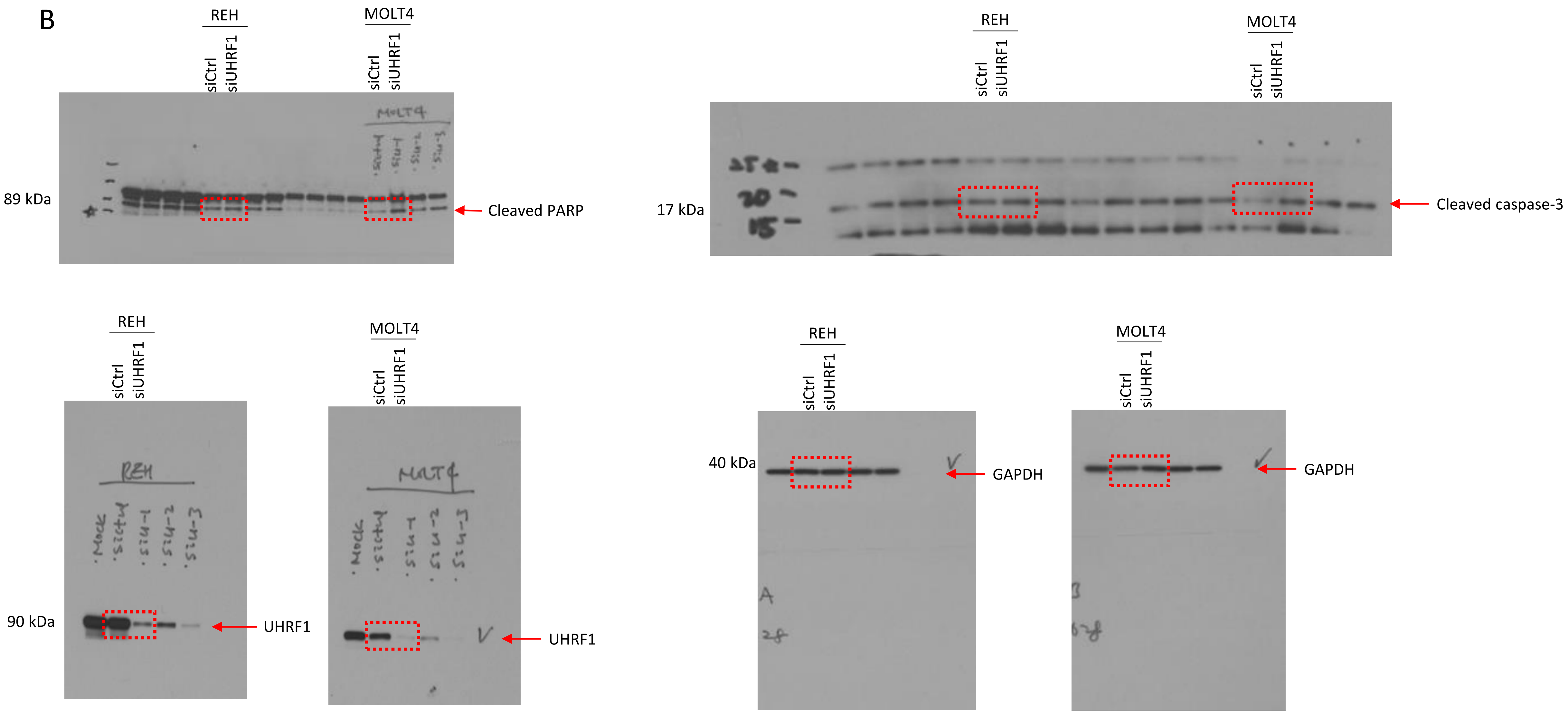
B
REH
siCtrl
siUHRF1
MOLT4
siCtrl
siUHRF1
89 kDa
Cleaved PARP
REH
siCtrl
siUHRF1
MOLT4
siCtrl
siUHRF1
17 kDa
Cleaved caspase-3
REH
siCtrl
siUHRF1
90 kDa
UHRF1
MOLT4
siCtrl
siUHRF1
UHRF1
REH
siCtrl
siUHRF1
40 kDa
GAPDH
MOLT4
siCtrl
siUHRF1
GAPDH

Figure 5 A and B.

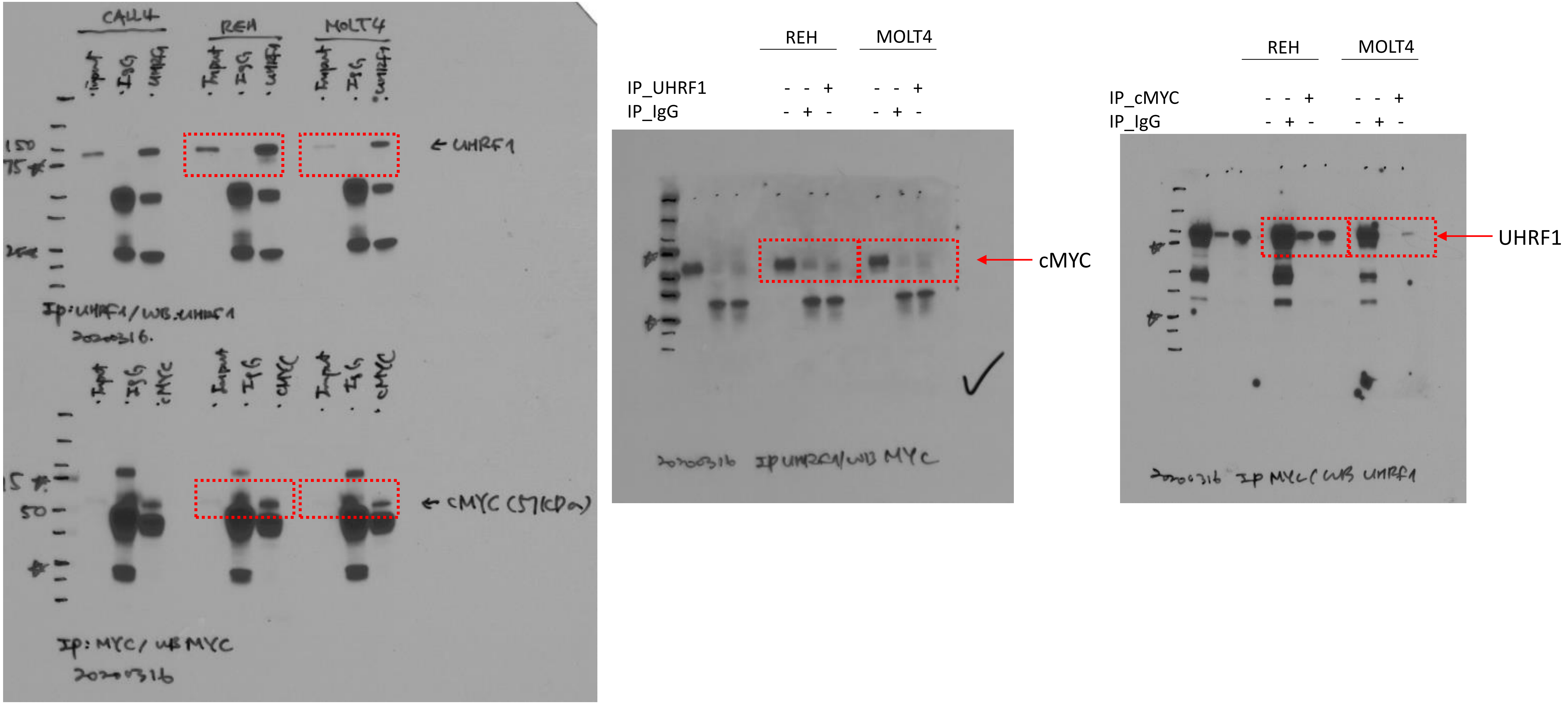

Figure A2.

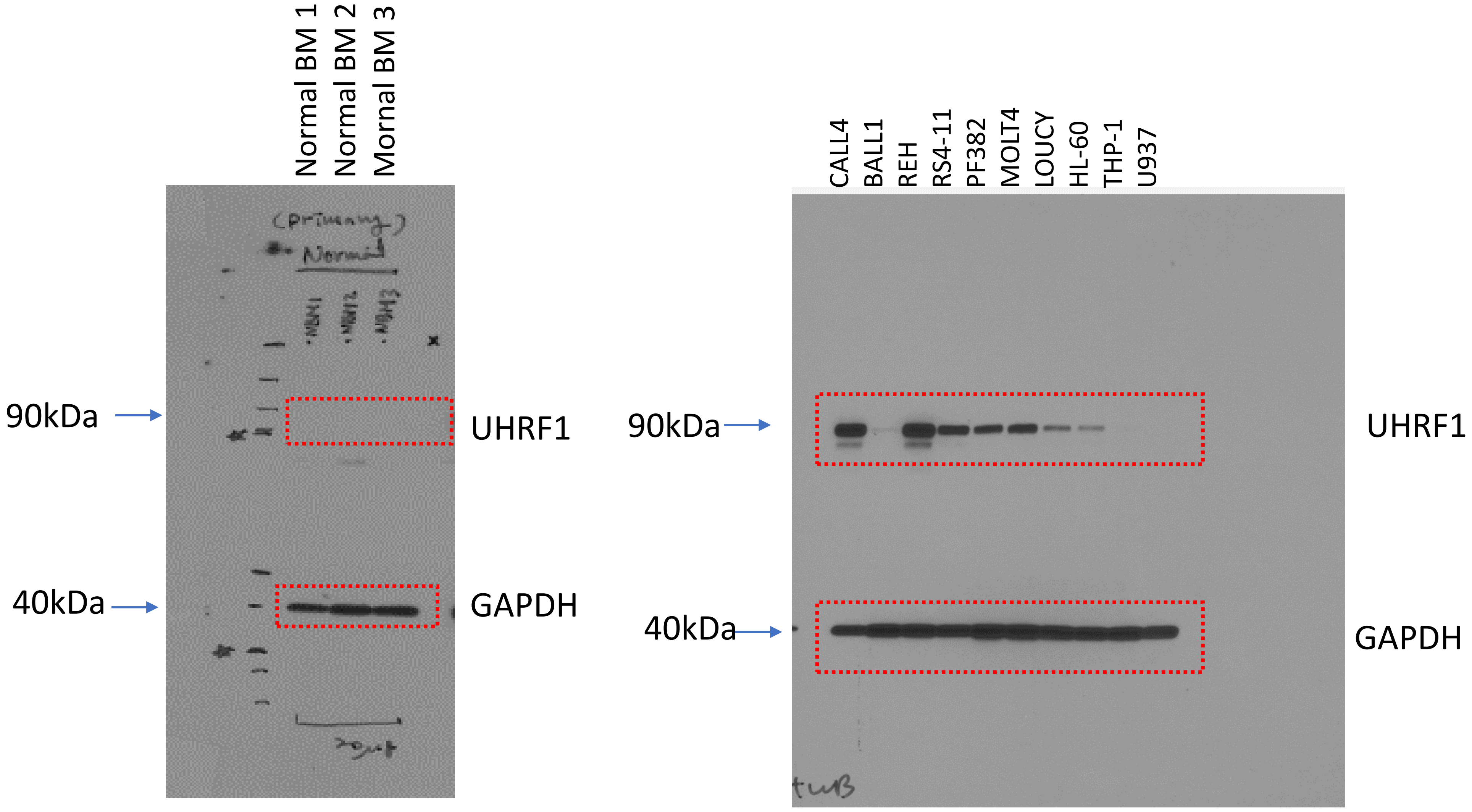
Normal BM 1
Normal BM 2
Mormal BM 3
90kDa
UHRF1
40kDa
GAPDH
CALL4
BALL1
REH
RS4-11
PF382
MOLT4
LOUCY
HL-60
THP-1
U937
90kDa
UHRF1
40kDa
GAPDH

Figure A4.

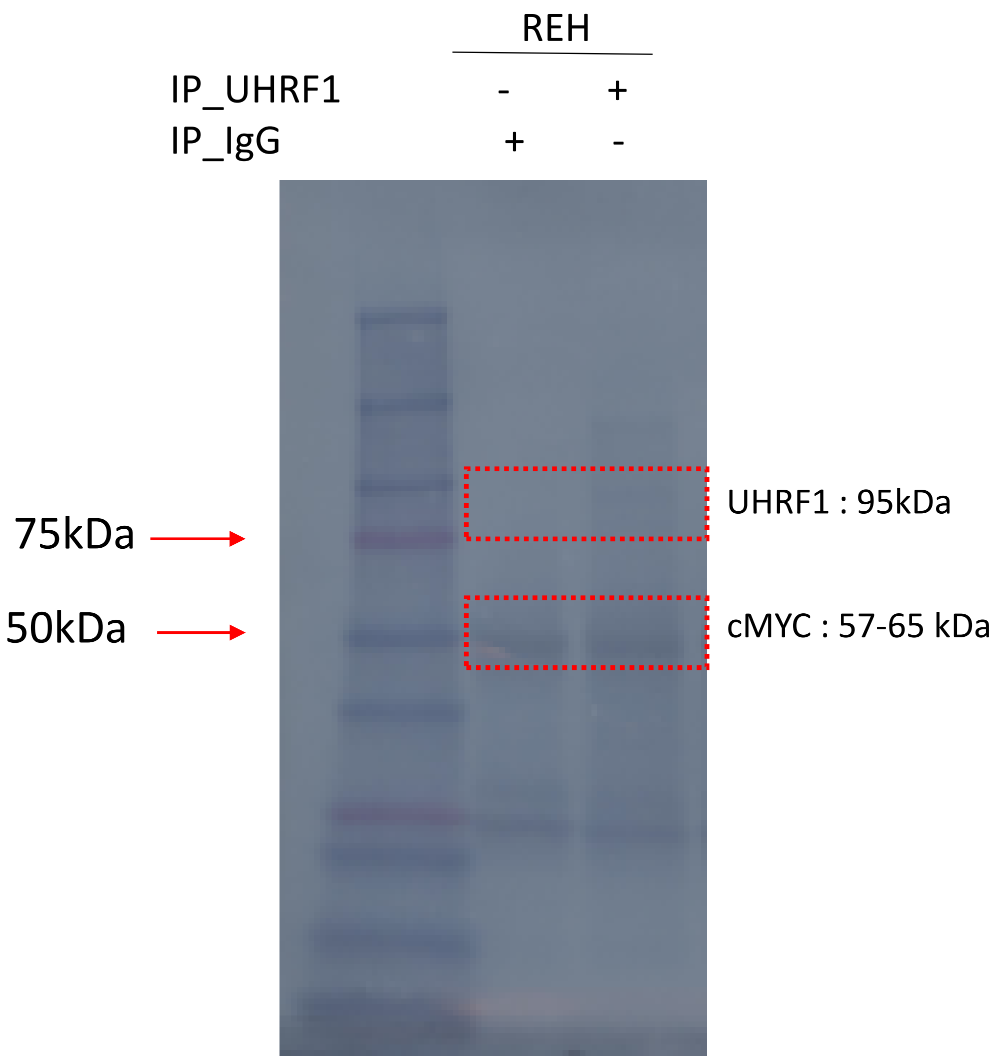

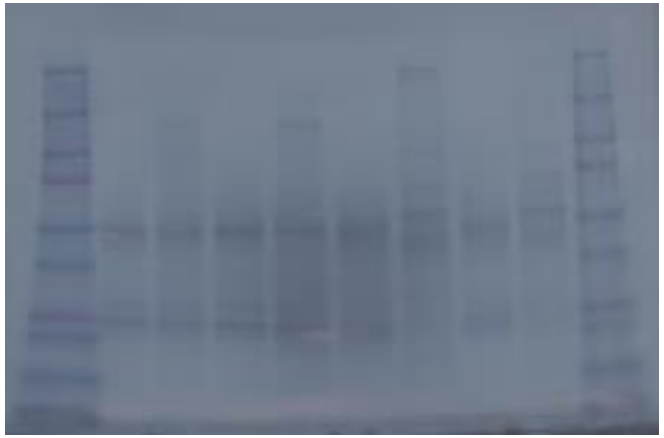

Supplement: Supplementary file 1 [file cancers-14-04262-s001.zip › cancers-1878176-supplementary.pdf]
